# Iodine density mapping for the diagnosis of acute bowel ischemia using fast kV-switching dual-energy CT

**DOI:** 10.1007/s00261-023-04097-4

**Published:** 2023-11-17

**Authors:** Jack Junchi Xu, Peter Sommer Ulriksen, Samir Jawad, Yecatarina Zincuk Rohde, Morten Sejer, Michael Patrick Achiam, Timothy Andrew Resch, Lars Lönn, Kristoffer Lindskov Hansen

**Affiliations:** 1 Department of Diagnostic Radiology, Copenhagen University Hospital, Rigshospitalet, 2100 Copenhagen, Denmark; 2 Department of Clinical Medicine, University of Copenhagen, 2100 Copenhagen, Denmark; 3 Department of Surgery and Transplantation, Copenhagen University Hospital, Rigshospitalet, 2100 Copenhagen, Denmark; 4 Department of Vascular Surgery, Copenhagen University Hospital, Rigshospitalet, 2100 Copenhagen, Denmark

**Keywords:** Computed tomography, Dual-energy CT, Bowel ischemia

## Abstract

**Purpose:**

To evaluate the diagnostic capabilities of a supplementary color ramped iodine density map compared to virtual monoenergetic images (VMIs) at 74 keV in the diagnosis of acute bowel ischemia (ABI).

**Methods:**

Data for this study were prospectively gathered and retrospectively evaluated. Patients referred to the Department of Diagnostic Radiology between October 2020 and August 2022 on the suspicion of ABI and underwent surgery < 12 h following fast kV-switching venous phase abdominal dual-energy CT (DECT) were consecutively included. Images were evaluated by two board-certified radiologists and two radiology residents. First round included only 74 keV VMIs resembling conventional 120 kVp images, and the second round included a supplementary iodine density map. Readers were asked to register presence of ABI as well as their confidence in their diagnosis based on a 5-point Likert scale. Sensitivity, specificity, positive predictive value (PPV), and negative predictive value (NPV) were calculated for each observer with the surgical findings as the gold-standard. McNemar’s and Wilcoxon signed-rank test were used to compare registrations and diagnostic confidence across assessment rounds.

**Results:**

A total of 29 patients resulting in 31 DECT scans were included. Fourteen cases of ischemic/necrotic bowel were reported following surgery. Sensitivity and NPV were decreased with the use of supplementary iodine map images compared to 120 kVp-like images without supplementary iodine map images for three of four observers (round 1 range: 71.4–92.9% and 78.0–94.8%; round 2 range: 57.1–78.6% and 70.1–83.3%, respectively), while specificity and PPV were increased for three of four observers (round 1 range: 64.7–94.1% and 67.4–93.1%; round 2 range: 88.2–94.1% and 73.8–91.1%, respectively). However, no significant difference in ABI diagnosis or diagnostic confidence was found (*p*-value range: 0.07–1.00 and 0.23–0.58, respectively).

**Conclusion:**

No significant difference for the diagnosis of ABI was found using supplementary iodine mapping. Our study may suggest a trend of increased specificity and decreased sensitivity, hence, the use of supplementary iodine mapping should be carefully considered.

## Introduction

Acute bowel ischemia (ABI) is a condition where a sudden reduction or interruption of the blood flow to the small or large intestine results in bowel ischemia and eventual necrosis and perforation [1]. ABI may be caused by arterial or venous occlusion, non-occlusive ischemia or bowel strangulation/obstruction [2]. ABI is a rare diagnosis constituting approximately 0.09–0.2% of surgical admissions [3]. However, due to high mortality rates associated with intervention of ABI (60–80%), the diagnosis of ABI has a high priority among abdominal surgeons [3, 4].

One of the most important prognostic factors for patient outcome is related to the duration of the intestinal hypoperfusion [5]. Although ABI due to arterial and venous occlusion as well as non-occlusive ischemia can be diagnosed with high sensitivity (93%) and specificity (96%) using multiphase CT, sensitivity for ABI secondary to obstruction or strangulation is associated with a significantly lower sensitivity (49%) [6]. Considering that small bowel obstruction (SBO) accounts for 12–16% of acute abdominal pain admissions, the all-cause sensitivity for ABI is presumably significantly lower than 90% [7].

Multiple CT features contribute to the diagnosis of ABI, however, one of the most specific signs for ABI is diminished enhancement of the bowel wall, which may be found in all causes for ABI [8–10]. The sensitivity of bowel wall hypo-enhancement may be as low as 48%, which can be explained by the difficulties associated with the identification of subtle differences in gray scale attenuation [9, 11].

With the emergence of dual-energy CT (DECT), several studies have indicated that iodine mapping and iodine quantification can aid in the diagnosis of ABI [12, 13]. DECT uses data from high and low energy photons to separate materials based on their atomic number. Due to the high atomic number of iodine, the attenuation of iodine contrast increases relatively more with decreased photon energy compared to other tissues in the body. DECT allows for the separation of iodine from soft tissue or water with the reconstruction of iodine maps, that can be a potential aid in various clinical tasks [14, 15].

In this study, we aimed to assess the diagnostic performance of supplementary iodine density map derived from DECT image acquisition compared to conventional CT images for the evaluation of ABI. Secondly, we investigated how iodine density maps affected the diagnostic performance of residents versus experienced abdominal radiologists. We hypothesized that DECT may increase the sensitivity, specificity, and the confidence of ABI diagnoses. The radiological findings with and without DECT iodine reconstructions were compared to surgical findings serving as the gold-standard.

## Methods

This study was approved by the Regional Committee on Health Research Ethics and the Regional Knowledge Centre on Data Protection Compliance (P-2020-663). Data were prospectively collected.

### Patients

Two main criteria were required for inclusion in the study. First, patients had to be referred to the Department of Diagnostic Radiology on the suspicion of ABI and undergo a venous phase DECT or multiphase ABI protocol including a venous phase DECT and a conventional non-contrast and arterial phase scan. Second, patients had to undergo abdominal surgery no more than 12 h following the image acquisition. Surgical findings were based on a visual and/or palpatory assessment of the bowel, and findings as stated in the surgical report were used as reference. Second look assessments were also included if available. Included patients were considered as independent samples.

Patients referred between October 2020 and August, 2022 were screened for inclusion. The same patient could be included multiple times if inclusion criteria were met. For these patients, statistical independence was assumed. Patient scans were excluded if image quality was severely affected by motion- or metal artifacts. Patient demographics and surgical findings were obtained through the regional electronic journal, Sundhedsplatformen (Epic Systems, Madison, Wisconsin, US). Dose reports including CT dose index volume (CTDIvol) and dose length product (DLP) were obtained within the local picture archiving and communication system (PACS).

### Scan parameters

All patients were scanned in a second-generation 256-slice CT (Revolution CT; GE Healthcare, Chicago, IL, USA). For the conventional non-contrast and arterial phase scan a kVp of 120 was used with a tube current between 80 and 600 mA (SmartmA) and a noise index of 13 and 14, respectively. For the venous phase acquisition, fast kV-switching (80/140 kV) was applied using Gemstone Spectral Imaging (GSI) Assist (GE Healthcare, Chicago, IL, USA) with a noise index of 14. Rotation speed, pitch, and beam width for all phases were set to 0.5 s, 0.992, and 80 mm, respectively.

Omnipaque 350 mg/mL (Iohexol, GE Healthcare, Chicago, IL, USA) was administered through an 18-gauge plastic cannula in the antecubital vein. Contrast volume was 1.4 mL/kg at a flow rate of 4 mL/s. The arterial and venous phase delay were set to 7 and 45 s following HU threshold triggering within the aorta at 100 HU.

### Image reconstruction

Images were reconstructed using an adaptive statistical iterative reconstruction (ASIR-V) algorithm with a blending factor of 60% [16]. A standard abdominal kernel was applied. The data file for the venous phase scan as well as 0.625 mm sliced images for all scan phases were transferred to the advanced workstation (AW) server for image analysis. DECT images were reconstructed at 74 kiloelectron volt (keV) to resemble 120 kVp images.

### Image analysis

Images were evaluated by four observers, two third year residents and two board-certified radiologists with 7 and > 20 years of clinical experience, respectively. Images were assessed on the AW server to blind observers from the list of previous examinations, referral text, radiology report and markers set by the reporting radiologist. All observers evaluated the images independently over two rounds. A gap of 6 weeks was set between the assessment of the last patient in the first round and first patient in the second round to blind observers from their previous assessments.

**Fig. 1:**
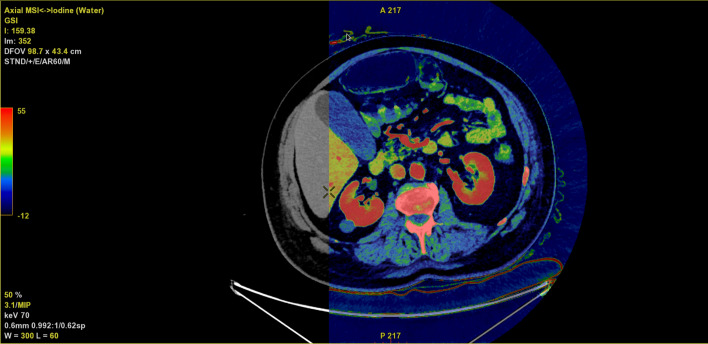
Axial image of the abdomen with an iodine overlay (colored map) on top of a virtual non-contrast image (underlying gray scale image). The color ramp ranging from − 12 to 55 (shown to the far left) represents the varying iodine concentrations in $\mathrm{\mu gI}/\mathrm{cm}$^3^

Round 1 included conventional 120 kVp-like images (venous phase and conventional non-contrast/arterial phase if available) and round 2 included iodine density reconstructions as well as the 120 kVp-like reconstructions presented in the first round. Each observer was given a patient list, which was intra- and inter-reader randomized by simple randomization across the two assessment rounds. A custom workflow within the AW server was setup for the second assessment round. This workflow included a virtual unenhanced (VUE) image overlayed by an iodine (water) map with a rainbow color ramp allowing the observer to freely overlay the iodine density map on top of the VUE image (Fig. 1). The color ramp provides a semiquantitative measure for the iodine concentration, which by default was set to − 12 to 55 $\mathrm{\mu gI}/\mathrm{cm}$^3^ based on previous experience at our institution. Observers were briefly (1 h) introduced to the AW server and how to navigate and adjust slice thickness, color ramp settings, window level, and multi-planar reconstruction prior to the assessment rounds.

For each assessment round, observers were asked whether they suspected ABI to be present or not. If ABI was suspected, the observers were asked to register the affected region(s) defined by the following list: duodenum, jejunum, ileum, small bowel, caecum, ascending colon, transverse colon, descending colon, sigmoid colon, and rectum. Furthermore, observers were asked to rate their diagnostic confidence on a five-point Likert scale (1 = ABI cannot be excluded, 3 = moderate diagnostic confidence for ABI, 5 = high diagnostic confidence for ABI) similar to a previous study by Lourenco et al. [12, 17]. This confidence rating was only registered for patients in which ABI was suspected.

### Statistical analysis

Sensitivity, specificity, negative predictive value (NPV), and positive predictive value (PPV) were calculated for each observer with and without DECT reconstructions. Furthermore, McNemar’s test was used to assess whether a significant difference was observed between the two assessment rounds, while Wilcoxon signed-rank test was used to evaluate changes in confidence rating for the same observer. A significance level of 0.05 was applied.

Inter-observer agreement was evaluated using kappa statistics (< 0 = poor; 0.0–0.20 = slight agreement; 0.21–0.40 = fair agreement; 0.41–0.60 = moderate agreement; 0.61–0.80 = substantial agreement; and 0.81–1.0 = almost perfect agreement) [18]. Statistical analyses were performed in R version 4.0.1 (R Foundation for Statistical Computing, Vienna, Austria) with RStudio version 1.2.1093 (RStudio, Boston, MA, USA).

## Results

**Fig. 2:**
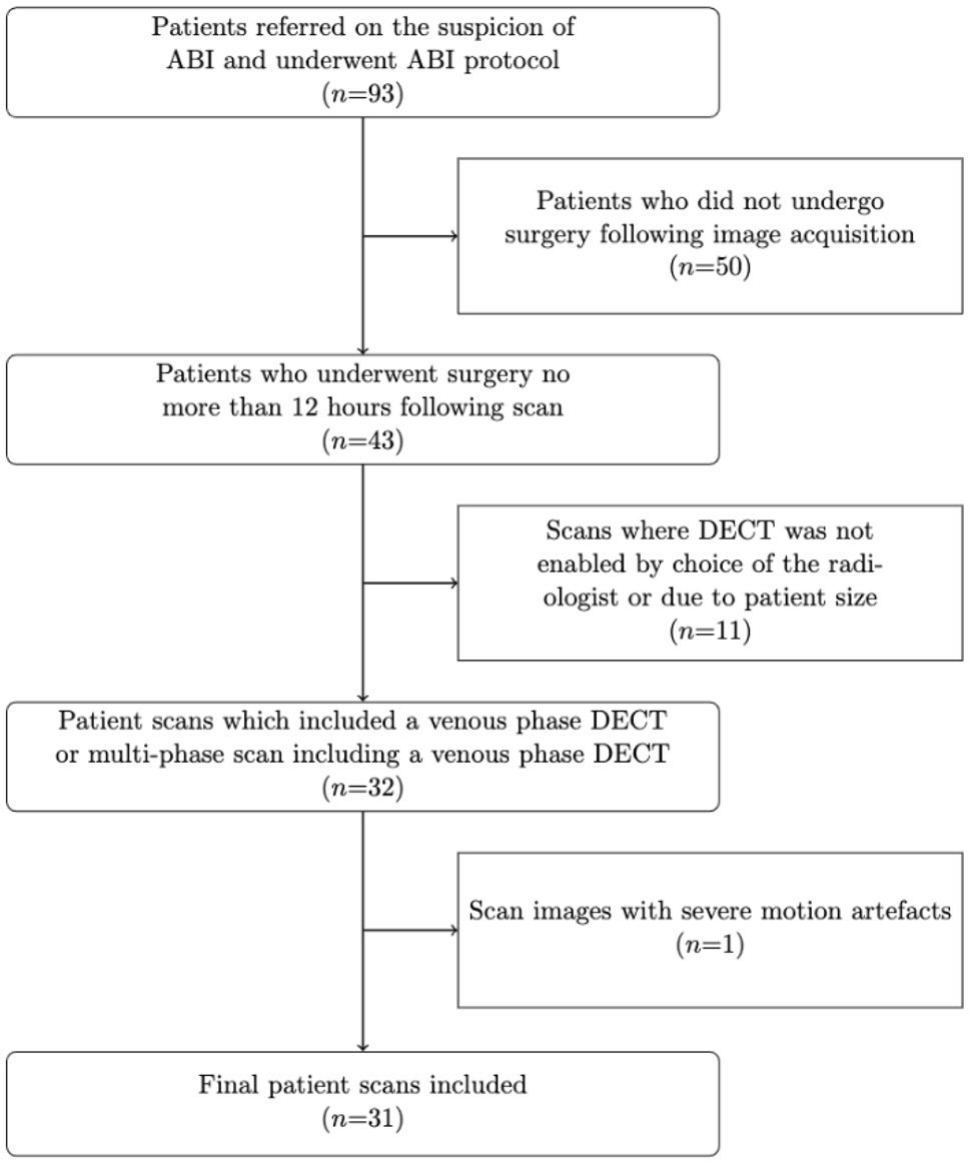
Flowchart of the patient inclusion process

Ninety-three patients underwent CT according to the ABI protocol, and a total of 29 patients, 16 men and 13 women (mean age 64 ± 12 years, range: 32–87 years), were included in this study (Fig. 2). Two patients were scanned and consequently operated twice with a separation time between first and second round of image acquisition and subsequent surgery of 4 and 18 days, respectively. This resulted in a total of 31 evaluated image datasets with matching surgical reports. Median time from image acquisition to surgical intervention (as defined by the registration time of the surgical report) was 4 h and 35 min. Fourteen patients were found to have ischemic/necrotic bowel following surgery and the mortality rate was 71% (*n* = 10). Affected bowel sections included the colon (*n* *=* *4*), small bowel (*n* *=* *4*), and mixed (*n* *=* *6*). No patients underwent second look operation following registered surgical procedures, however, one of the included surgeries was a second look operation. Eight patients underwent venous phase DECT (mean CTDIvol = 11.8 ± 1.5 and mean DLP 132.1 ± 8.0), while the remaining 23 patients underwent multi-phase scan (mean CTDIvol = 30.0 ± 6.8 and mean DLP 1742.4 ± 485.0).

### Subjective image interpretation

For all observers, sensitivity either was maintained or decreased using supplementary iodine map: (round 1 range: 71.4–92.9%; round 2 range: 57.1–78.6%), while specificity was increased for three observers (round 1 range: 64.7–94.1%; round 2 range: 88.2–94.1%). Likewise, PPV was increased for the same three observers (round 1 range: 67.4–93.1%; round 2 range: 73.8–91.1%), while NPV was maintained or decreased (round 1 range: 78.0–94.8%; round 2 range: 70.1–83.3%). An overview of the performance of the four observers in the two rounds is shown in Table 1.

**Table 1:** Sensitivity, specificity, positive predictive value (PPV), and negative predictive value (NPV) with respective 95% confidence intervals (CI) for all observers for conventional and iodine map images

	Sensitivity* (95%CI; TP/[TP + FP])	Specificity* (95%CI; TN/[TN + FP])	PPV* (95%CI; TP/[TP + FP])	NPV* (95%CI; TN/[TN + FN])	Average confidence
Conventional 120 kVp-like images					
Radiologist 1	85.7 (56–100; 12/14)	64.7 (46–83; 11/17)	67.4 (45–88; 12/18)	84.6.0 (65–100; 11/13)	3.9
Radiologist 2	78.6 (52–100; 11/14)	88.2 (57–100; 15/17)	84.6 (65–100; 11/13)	83.3 (66–100; 15/18)	4.2
Resident 1	71.4 (48–94; 10/14)	82.4 (54–100; 14;17)	76.9 (54–100; 10/13)	78.0 (59–96; 14/18)	3.4
Resident 2	92.9 (60–100; 13/14)	94.1 (61–100; 16/17)	92.9 (79–100; 13/14)	94.1 (83–100; 16/17)	4.0
Conventional 120 kVp-like images + Iodine map					
Radiologist 1	57.1 (44–70; 8/14)	82.4 (54–100; 14/17)	72.7 (46–99; 8/11)	70.0 (47–87; 14/20)	4.3
Radiologist 2	71.4 (48–94; 10/14)	94.1 (83–100; 16/17)	90.9 (74–100; 10/11)	80.0 (67–100; 16/20)	4.0
Resident 1	71.4 (48–94; 10/14)	88.2 (78–98; 15/17)	84.6 (62–100; 11/13)	78.9 (59–95; 15/19)	3.2
Resident 2	78.6 (52–100; 11/14)	88.2 (78–98; 15/17)	84.6 (65–100; 11/13)	83.3 (75–100; 15/18)	4.2

*TP* true positive, *TN* true negative, *FP* false positive, *FN* false negative

*Values given in percentages

McNemars test demonstrated no significant difference between the two evaluation rounds for all observers (*p* > 0.05; range: 0.1–1.0, Table 2). Additionally, no significant difference was found in the confidence rating across the two rounds for all observers (*p* > 0.2; range: 0.2–0.6, Table 2). For the registered bowel segment(s), 96% of the registrations correlated with the affected segment(s) as stated in the surgical report. Only two registrations by resident 2 in round 1 did not match surgical findings: One registration of ABI suspicion in the descending colon was surgically identified to the caecum and terminal ileum, and a second registration within the caecum and ascending colon was identified to the small bowel.

**Table 2:** Comparison values across the evaluation rounds for the diagnosis of ABI (McNemar) and the observer rated confidence (Wilcoxon)

	Diagnosis of ABI (McNemar) *p*-value	Observer rated confidence (Wilcoxon) *p*-value
Radiologist 1	0.07	0.23
Radiologist 2	0.48	0.57
Resident 1	1.0	0.57
Resident 2	1.0	0.58

Inter-observer agreement showed overall substantial agreement for 10/12 comparisons for conventional and iodine map evaluations (range: 0.59–0.93 and 0.49–0.87, respectively, Table 3). Two remaining comparisons between radiologist 1 and resident 2 demonstrated moderate agreement (κ = 0.59 and 0.49, Table 3).

**Table 3:** Interobserver agreement between all observers for conventional and iodine map images

	Conventional images	Iodine map
Rad1/Rad2	0.72	0.68
Rad1/Res1	0.79	0.69
Rad1/Res2	0.59	0.49
Rad2/Res1	0.93	0.87
Rad2/Res2	0.86	0.80
Res1/Res2	0.80	0.67

*Rad1* Radiologist 1, *Rad2* Radiologist 2, *Res1* Resident 1, *Res2* Resident 2

## Discussion

Overall, we found no significant difference between the use of conventional images compared to conventional images combined with iodine maps. This study found that specificity may be increased using iodine map images as a supplementary reconstruction to conventional multiphase images, however, a decrease in sensitivity was also identified. Our findings suggest that systematic use of iodine density maps may lead to misdiagnosis of ABI when compared to surgical findings.

Few studies have evaluated the clinical benefits of using iodine maps as a supplementary reconstruction to conventional reconstructions for the diagnosis of ABI. To our knowledge, only one study by Lourenco et al. evaluated the clinical performance of iodine maps, demonstrating a maintained or increased sensitivity and specificity as well as increased confidence in the diagnosis of ABI for one of two observers [12].

**Fig. 3:**
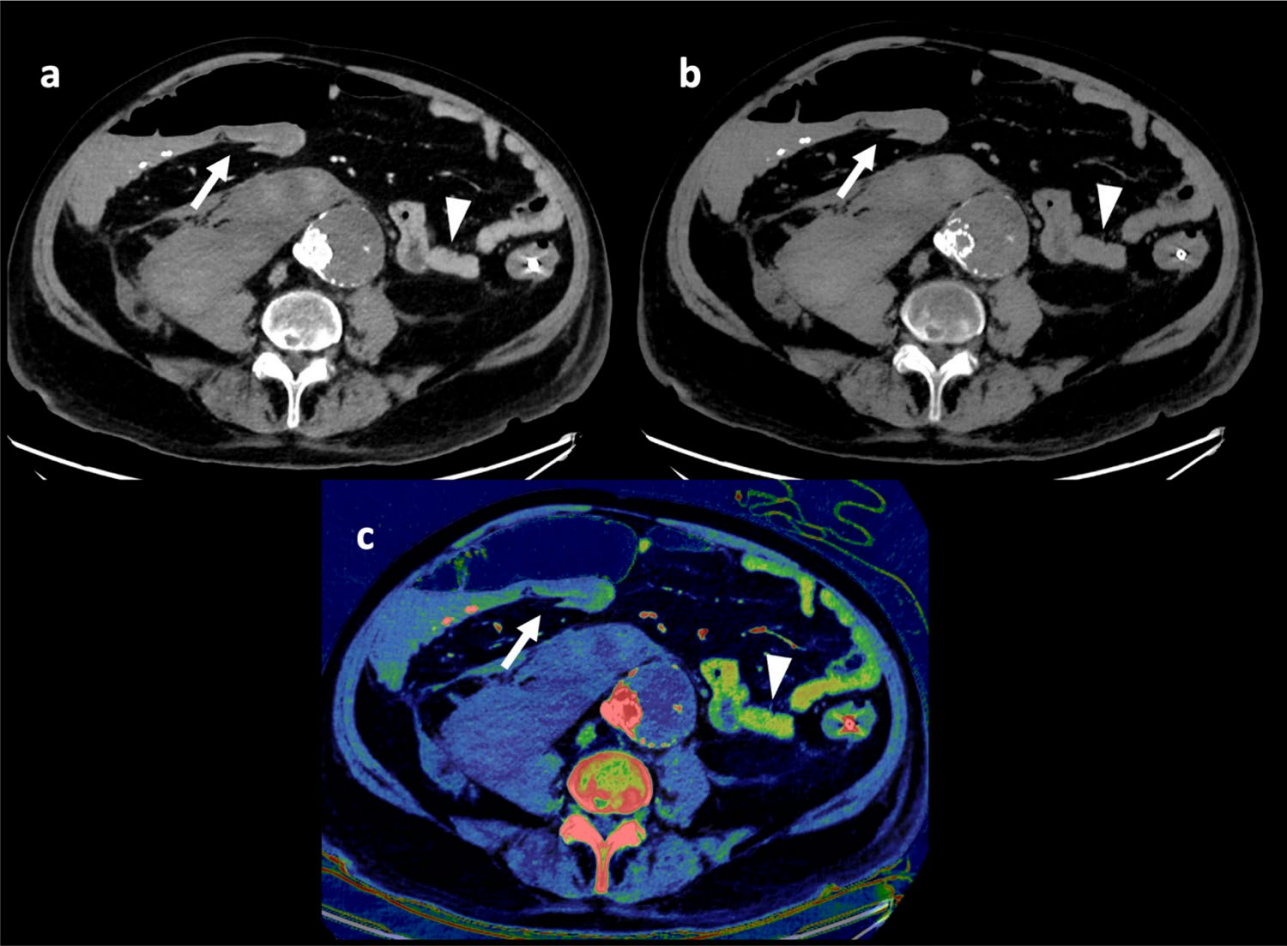
A patient with surgically confirmed ischemic/necrotic caecum, ascending colon, and proximal portion of the transverse colon. Three of four observers evaluated the colon to be ischemic (arrow) in the 120 kVp-like images (**a**) during the first evaluation round. However, during the second evaluation round, with the iodine map (**c**) and underlying virtual unenhanced (VUE) image (**b**), only one of four observers deemed the bowel to be ischemic. In **c** the ischemic section of the colon may be interpreted as perfused (arrow) when compared to other vital bowel segments (arrowhead). Upon closer inspection of the VUE images, it is noticeable that the ischemic bowel maintains some bowel wall attenuation (arrow) compared to the non-ischemic bowel (arrowhead) suggesting a paired effect. Additionally, pseudo hyperenhancement due to an adjacent air distended bowel segment, may also contribute to the enhancement seen on the iodine map

These findings were only partially reproduced in our study, as we identified increased specificity and PPV for three of four observers, suggesting a decreased false positive rate. Following round two, the average specificity and PPV increased from 82.4 to 88.2% and 80.8 to 83.5%, respectively. This may be attributed to the improved visualization of bowel wall perfusion. Sensitivity, however, was decreased for three of four observers using iodine maps resulting in an average reduction from 82.2 to 69.6% following round two. Additionally, no significant difference was found in the confidence of ABI diagnosis for any observers. Interestingly, for two of four observers the average confidence rating decreased with the use of iodine mapping, demonstrating that there were cases where the iodine map rendered the observer less confident or led to a misdiagnosis compared to the primary evaluation. Several factors may explain this observation. First, natural intra-reader variability across time cannot be excluded [19]. Second, limitations related to the underlying physics of DECT, and material decomposition may affect the observational outcome [20]. An example may be the understanding of paired Compton and photoelectric contribution for two material decomposition maps, where materials such as calcium or similar materials can demonstrate a paired effect (Fig. 3). In these cases, the same material may attenuate in both iodine(water) and VUE images. Additionally, several other known artifacts have been described in relation to bowel wall assessment using iodine density maps such as pseudo hyperenhancement caused by air-filled bowel segments, peristalsis related artifacts, and streak artifacts from metal [20]. Third, the added number of reconstructions may decrease the overall assessment thoroughness leading to missed diagnoses, which also was suggested by a recent study [21].

**Fig. 4:**
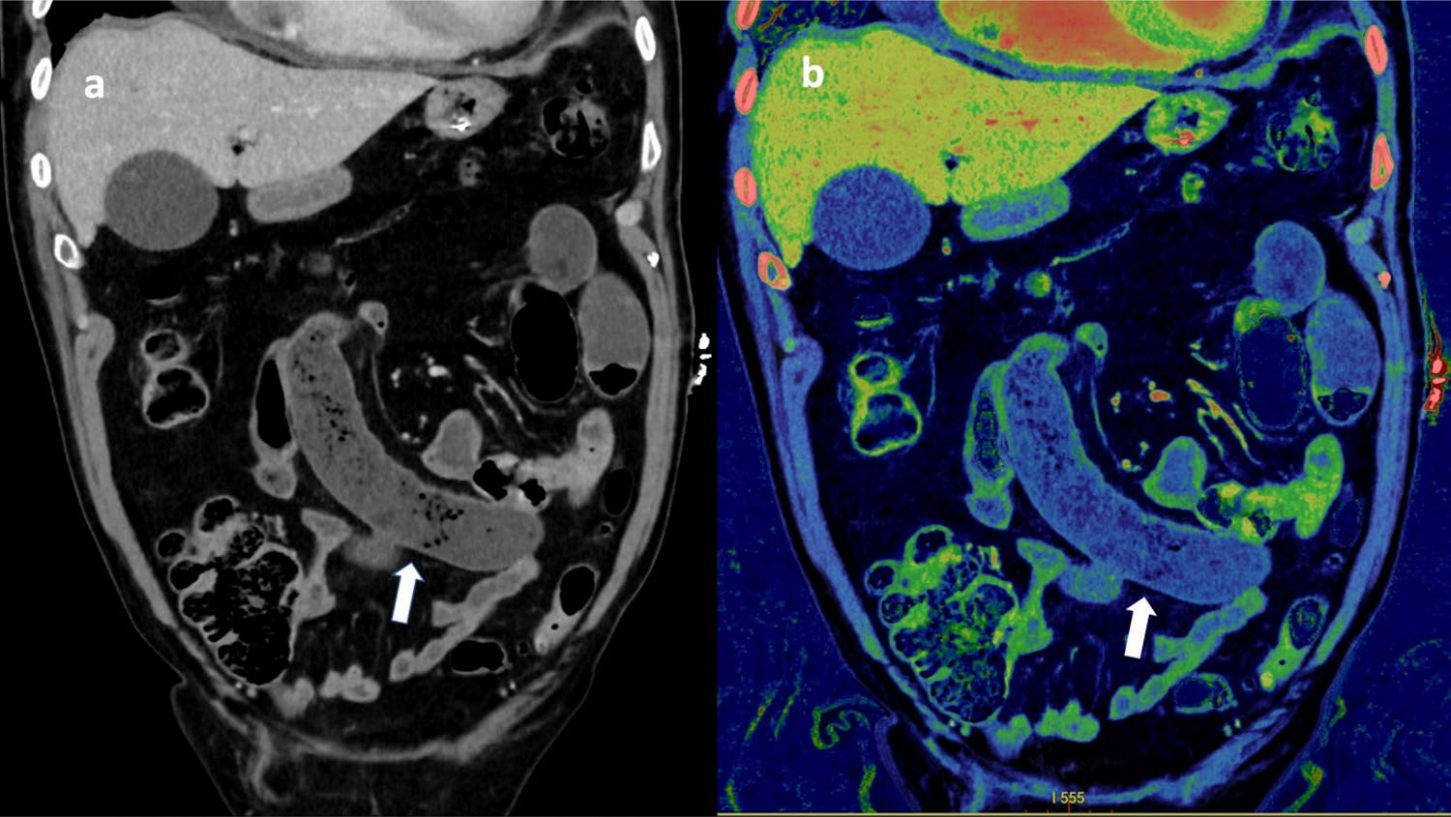
A patient with surgically confirmed small bowel ischemia due to closed loop bowel obstruction (arrow). Despite some attenuation in the bowel wall seen on the conventional 120 kVp-like image (**a**), one resident observer suspected ischemia and became more confident using iodine map (**b**). Additionally, one radiologist only reported ischemia using the iodine map

**Fig. 5:**
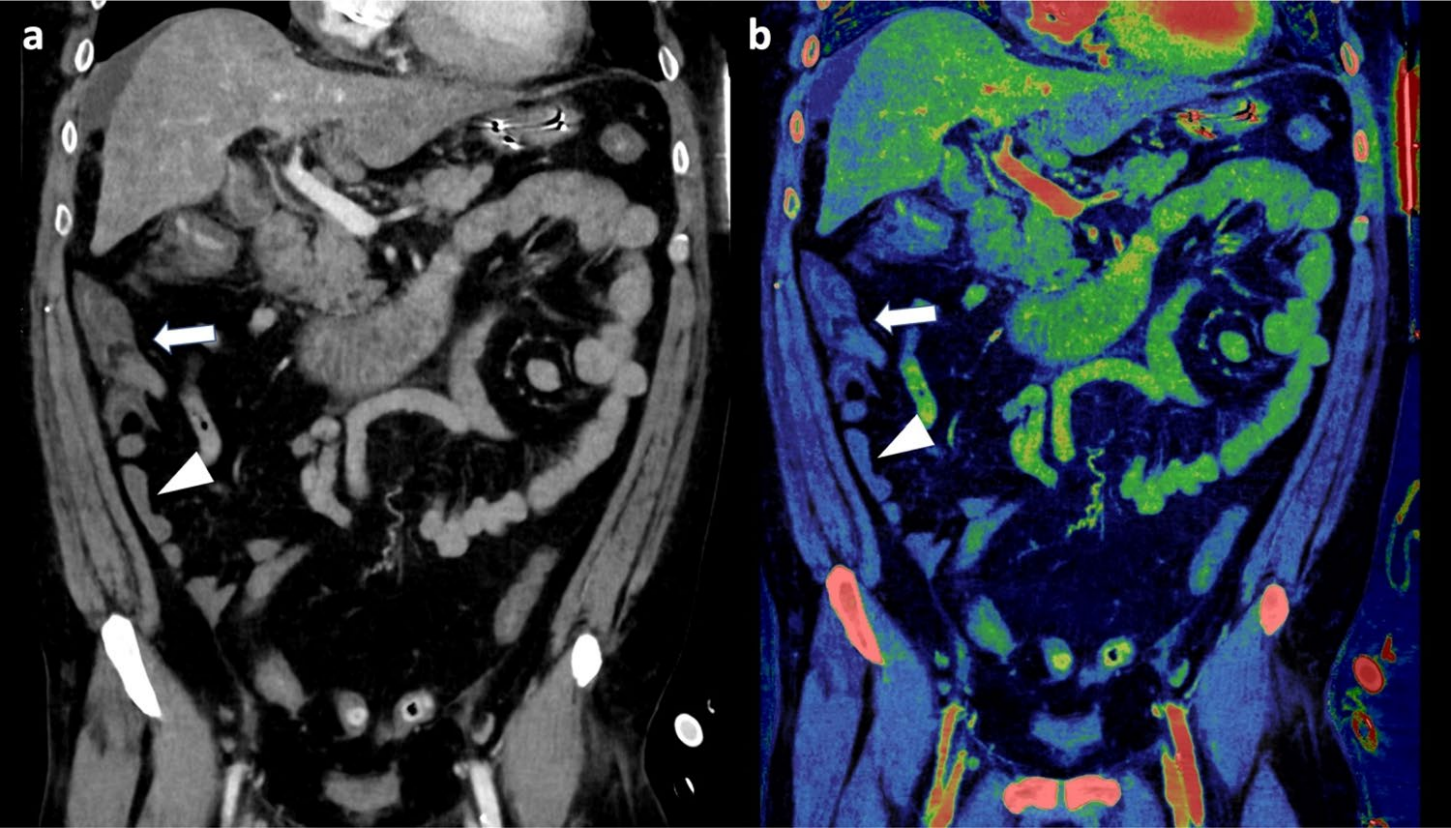
A case for which all observers suspected bowel ischemia located to the ileum (arrowhead), caecum and ascending colon (arrow). A confidence rating of three was given by three observers while the last observer rated four in the 120 kVp-like images (**a**). Following evaluation with iodine mapping (**b**), the three observers who initially gave a confidence rating of three now confidently diagnosed ischemia with an increased confidence rating of five, while the last observer maintained a confidence rating of four. However, in this case the surgical report found no signs of ischemia

Previous studies demonstrated increased conspicuity of ABI with DECT reconstructions such as iodine mapping [13]. This was also the case for a few patients in this study (Fig. 4), though most ischemic cases seemed to be easily identified in the conventional 120 kVp-like images. Furthermore, there were cases where all observers reported strong suspicion of ischemia with increased confidence using the iodine map, but where the surgical findings found no signs of ischemia (Fig. 5). As this particular patient survived and did not undergo second look operation, the mismatch between radiological and surgical findings could be explained by temporary hypoperfusion and reperfusion.

No significant difference was found between the reporting of resident level physicians and experienced radiologists in this study. The overall most correct evaluation for suspected ABI diagnosis was performed by resident 2 (Table 1). However, it is also relevant to add that the only two bowel segment registrations, which did not match the surgical findings, was registered by resident 2 during the first evaluation round with low confidence for both cases.

In this study, two patients were included twice, which may cause dependency concerns. We performed a Post-hoc sensitivity analysis (not shown), where the extra datasets from the two patients were excluded from the McNemar and Wilcoxon analyses. This did not significantly affect the results, and for this reason paired datasets were included in the presented analyses.

It is not evident from this study that iodine mapping using a semi-quantitative color scale improves the diagnosis of ABI. With the increasing workload for radiologists, the approach of evaluating all patients with iodine maps and DECT reconstructions may not be sensible [22]. This may be time inefficient and as demonstrated in this study lead to reduced sensitivity, which is crucial in the diagnosis of ABI. An emphasis on the technical aspects as well as the limitations of DECT may benefit radiologists and radiology residents to better assess on a case-to-case basis, whether DECT reconstructions may or may not be of use.

There are certain limitations to consider in this study. Firstly, although observers were blinded to the surgical report, they were aware that the surgical findings would be the gold standard. This contributes to a reporting bias as observers may expect more patients to have ischemia. As previous review articles have suggested, the use of DECT and iodine density maps in ABI diagnosis are heavily related to the increased conspicuity [23, 24]. In a study where identification of ABI is the main focus, these benefits may be less pronounced and thus may be more beneficial in cases where ABI is not the primary referral concern.

Secondly, this study only included 29 patients. Despite having a good distribution of surgically confirmed ischemia vs. no ischemia patients, a larger patient population with a bigger variety of negative and positive findings would have contributed to the statistical power of this study [23, 25]. Thirdly, although all observers had some experience with DECT reconstructions, no effort was made to ensure an understanding of the limitations and pitfalls of DECT prior to the evaluation. For future studies, a tailored training session should be considered for all observers to ensure a basic understanding of the technical limitations of DECT.

Lastly, surgical findings are not always accurate in predicting bowel perfusion and viability, as previous studies have suggested [26]. Future studies may benefit from including the histological findings as a gold standard reference.

## Conclusion

Our study found no significant difference when adding supplementary iodine map to conventional gray scale images in the diagnosis of ABI. This is the first study to include consecutive patients prospectively using surgical findings as reference standard, and the study outlines some of the challenges and potential pitfalls of using iodine maps in the diagnosis of ABI.
